# Diode Laser Therapy for Radiation-Induced Vascular Ectasia: Long-Term Results and Cost Analysis

**DOI:** 10.3390/life13041025

**Published:** 2023-04-16

**Authors:** Lino Polese, Emilia Giugliano, Roberto Cadrobbi, Deris Gianni Boemo

**Affiliations:** 1 First Surgical Unit, Department of Surgery, Oncology and Gastroenterology, University of Padova, 35128 Padova, Italy; giugliano.emilia@gmail.com (E.G.); roberto.cadrobbi@aopd.veneto.it (R.C.); 2 Medical Direction Unit, Padova University Hospital, 35128 Padova, Italy; derisgianni.boemo@aopd.veneto.it

**Keywords:** diode laser, radiation proctitis, vascular ectasia, rectum, radiofrequency ablation

## Abstract

Background: Collateral damage to surrounding healthy tissues has been reported in patients who undergo radiation therapy for pelvic malignancies. This study aimed to evaluate the safety, efficacy and cost efficiency of endoscopic diode laser therapy in patients diagnosed with chronic radiation proctitis (CRP). Methods: The data of 24 patients (median age 78, range 67–90 years) who presented rectal bleeding and were diagnosed with CRP after undergoing high-dose radiotherapy for prostatic cancer and underwent diode laser therapy were evaluated retrospectively. Non-contact fibers were used in the patients who underwent the procedure without sedation in an outpatient setting. Results: The patients underwent a median of two sessions; overall, a mean of 1591 J of laser energy per session was used. No complications were noted during or after the procedures. Bleeding was completely resolved in 21/24 (88%) patients, and two patients showed improvement (96%). It was not necessary to suspend antiplatelet (six patients) or anticoagulant (four patients) therapy during the treatment course. The mean cost per session was EUR 473.4. Conclusions: The study findings demonstrated that endoscopic non-contact diode laser treatment in CRP patients is safe, effective and cost efficient. For this procedure, antiplatelet and anticoagulant therapy suspension, intraprocedural sedation and hospital admission are not required.

## 1. Introduction

Although radiation therapy has been found to be an effective form of local tumor control, collateral damage to surrounding healthy tissues has been reported. Often used to treat pelvic malignancies, radiation therapy can, in fact, cause adverse effects on nearby organs. The rectum is at particular risk for injury during radiation therapy because of its fixed position in the pelvis. Chronic radiation proctitis (CRP) or proctopathy, for example, is a known complication associated to radiation therapy used to treat prostate cancer [1]. Its symptoms include bloody stools, diarrhea, fecal urgency, mucous discharge and tenesmus.

Rectal bleeding, which is the most common manifestation of CRP, occurs in more than 80% of patients suffering from the condition. Symptoms may present months or even years after radiotherapy due to the rupture of radiation-induced telangiectasias and friable ischemic mucosa [2]. Superficial vascular lesions gradually develop because of progressive epithelial atrophy and fibrosis associated with obliterative endarteritis and concomitant neovascularization. In any case, persistent rectal bleeding can lead to deficiency anemia that may require hospitalization and repeated blood transfusions [3].

Endoscopic findings of CRP are mucosal pallor, telangiectasias, spontaneous hemorrhage, edema and friability. The mechanism inducing abnormal angiogenesis in the superficial layer of the lamina propria but not in the submucosal one is not completely clear, but the result is easy bleeding from damaged blood vessels, frequently leading to anemia that may require hospitalization and multiple blood transfusions. The rectal mucosa may also be inflamed. What is known is that irradiated tissue usually has some degree of residual endothelial cell injury and endarteritis, which causes atrophy, fibrosis and poor tissue repair.

According to Takeuchi et al. [4], radiation proctitis may be caused by a complicated interaction between cytokines and the damaged tissue. Firstly, matrix metalloproteinase-8 (MMP-8) and urokinase-type plasminogen activator (uPA) produced by infiltrating B lymphocytes degrade the extracellular matrix and basement membrane to provide space for angiogenesis. Meanwhile, rectal epithelial cells and fibroblasts produce angiogenin and fibroblast grow factor 1 (FGF1) that stimulate endothelial cells to proliferate. These processes may continue for years after radiation therapy has been utilized because the mRNA level of endoglein, MMP-8 and FGF1 remains high after irradiation. The results of the study by those investigators were, however, unable to uncover the molecular mechanism underlying radiation-induced proctitis, or how to prevent or treat it.

According to Mahmood et al. [5], there are two distinct clinical entities linked to chronic radiation-induced damage to the rectum: radiation-associated vascular ectasias (RAVE) and CRP. The former, which seems to be characterized by the presence of vascular ectasias rather than fibrosis and ischemia, presents with rectal bleeding and chronic blood loss and anemia. The latter, which is characterized by epithelial damage to the rectum, presents with fecal urgency, changes in stool caliber and consistency, and increased mucus. In severe cases, fistulas and strictures may develop.

CRP should be suspected in any patient who has undergone pelvic radiotherapy and presents with the symptoms mentioned above. Endoscopy is important to confirm the diagnosis and exclude other causes of proctitis or malignancy. Endoscopy is also important to determine the extent and severity of CRP. Histology is not useful for diagnosis and biopsy is only justified when a malignancy is suspected. For diagnosis, assessment of severity, prognosis and response to treatment, different scoring systems are followed. They are based on symptoms, endoscopic findings, or both. According to Zinicola et al., [6] the disease can be classified according to the presence of fresh blood, the telangiectasia distribution and the surface area involved.

Several therapeutic options for radiation-induced vascular ectasia (RAVE), including lifestyle changes and nutrition, pharmaceutical therapy, rectal sucralfate enemas, topical formalin applications, and endoscopic and surgical procedures, have been utilized. The outcome of some of these treatments has been disappointing [7], and endoscopic management has become increasingly popular. A limit of many endoscopic techniques is still represented by the onset of necrosis of the treated tissue, which can result in ulcerations, with a risk of bleeding recurrence. Endoscopic diode laser therapy is a relatively new endoscopic modality for the treatment of CRP [8]. The 940 nm diode laser is characterized by its affinity for hemoglobin molecules and good coagulation properties. In particular, using non-contact fibers, it is possible to coagulate the vascular lesions with a low risk of tissue necrosis and mucosal erosions. This feature makes the treatment particularly safe and carries less risk of ulcerations. Consequently, the diode laser has been found to be a safe and effective treatment option for vascular ectasia [9].

The present study aims to evaluate the safety and cost-efficiency of diode laser therapy used to treat patients with bleeding CRP in a tertiary hospital setting.

## 2. Materials and Methods

**Figure 1:**
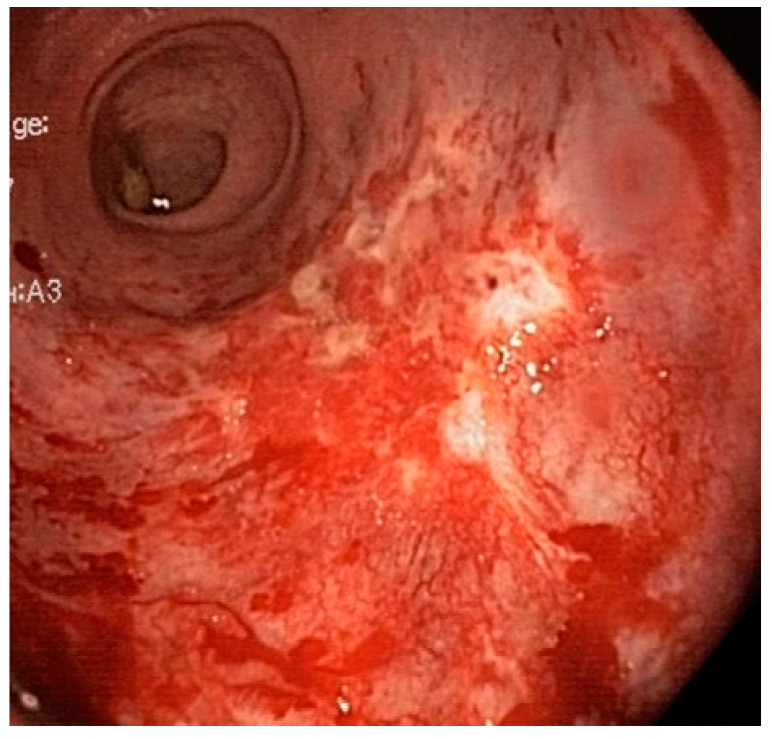
The affected rectum with RAVE.

This study, which is a retrospective analysis of prospectively collected data, was approved by the IRB and carried out in accordance with the principles of the Helsinki Declaration [10]. All the patients who underwent diode laser therapy for CRP at the University of Padova Medical Center between 2010 and 2021 were enrolled for the study. The study’s inclusion criteria were an endoscopic diagnosis of RAVE after radiotherapy to treat a pelvic neoplasm, and rectal bleeding, regardless of hemoglobin values presenting more than 3 months after radiation therapy [11]. Patients were excluded from the study if the rectal bleeding was caused by conditions other than CRP. The patients’ hospital records were reviewed; the severity of the CRP was graded on the basis of the endoscopic images and reports depending on the following criteria: the presence of fresh blood, the telangiectasia distribution and the surface area involved [6] (Figure 1).

### 2.1. Laser Therapy 

**Figure 2:**
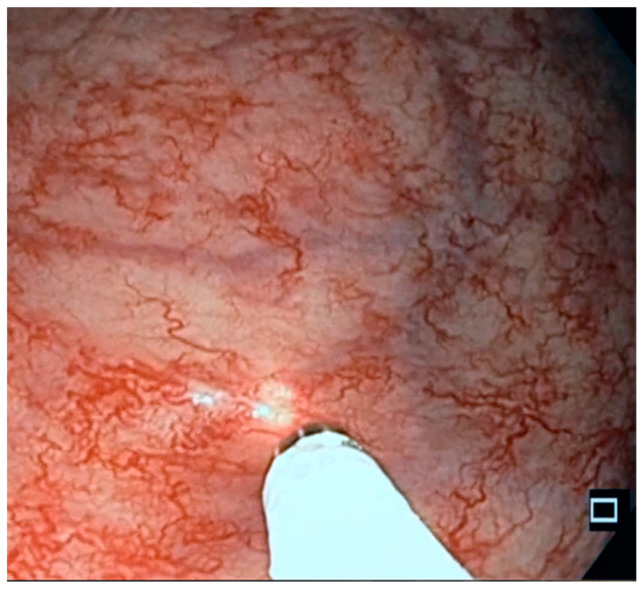
Diode laser treatment of RAVE. The diode laser is applied to the mucosa until the tissue is whitened. The exposure time is about 1 s.

All the patients signed informed consent statements and underwent endoscopic diode laser therapy without sedation, after the rectum was cleansed via two micro-enemas. It is known that the procedure is not painful and generally well tolerated. Conventional endoscopes and a high-power diode laser (Medilas D. Multibeam Dornier MedTech, France; maximum power 60 W; wave length 940 nm) were used because they have good coagulation properties [12] and a low penetration depth with respect to the Nd:YAG laser. Diode lasers are compact, lightweight units [13]. Vascular lesions were treated with diode lasers utilizing non-contact fibers at a 30 W power setting in continuous fibertom mode. (Figure 2). Disposable standard-lightguides with a nozzle tip of 1.8 mm were used. The diameter of the core fiber is 600 µm, and the length 3.5 m.

The diode laser was applied to the vascular lesions until the mucosa was whitened, avoiding greater damage and tissue necrosis.

In this way, avoiding the onset of erosions and ulcers, the patients were able to continue taking their oral anticoagulant or antiplatelet therapy (See Video S1).

**Figure 3:**
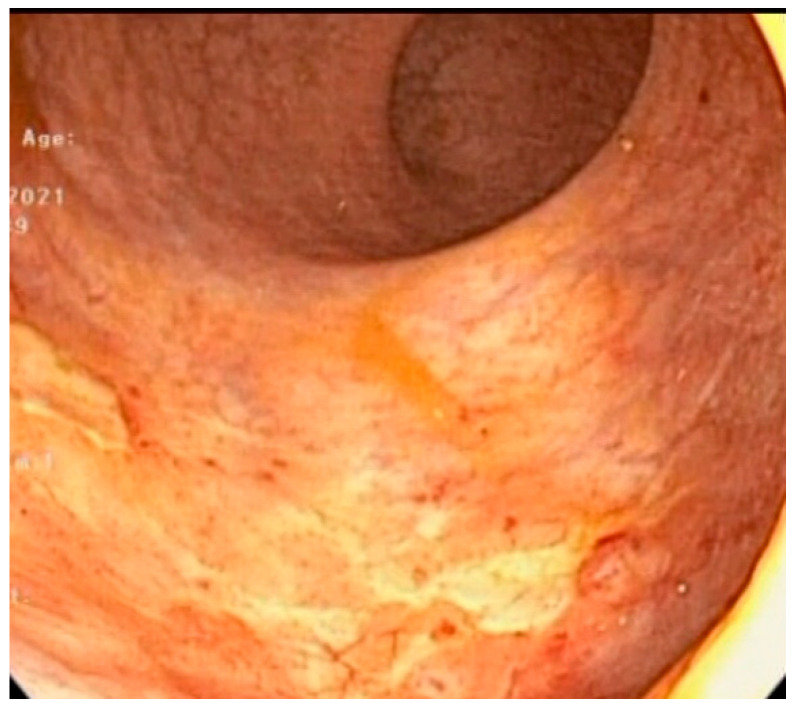
Diode laser: result at treatment conclusion.

Diode laser sessions were scheduled monthly, as previously reported [8], until complete healing of the RAVE (Figure 3) and complete remission of bleeding was obtained. Patients underwent a follow-up visit with rigid anoscopy and rectoscopy 3 months after the end of treatment. A blood test to confirm the improvement of anemia was also suggested 3 months after treatment.

All the patients were contacted by telephone to obtain information about their long-term follow-up.

The treatment was considered successful when there was a complete cessation of bleeding; a significant reduction in the number of bleedings (from daily to occasional) was considered an improvement.

### 2.2. Cost Analysis

The cost of the disposable materials and equipment used during the diode laser sessions was analyzed.

### 2.3. Statistical Analysis

Data are presented as mean ± standard deviation. The t test was used to compare the pre-treatment and post-treatment hemoglobin values. A *p* of <0.05 was considered significant.

## 3. Results

Twenty-four patients (median age 78, range 67–90 years) who were treated with diode laser therapy for RAVE between January 2010 and December 2021 were enrolled in the study. All the patients underwent radiotherapy because of prostate cancer.

All the patients had active overt rectal bleeding. Ten patients presented to the emergency department for bleeding and anemia and eight patients required hospitalization.

Six had received blood transfusions and 12 patients were on iron therapy.

Ten patients were taking medications for cardiovascular disease: five patients were receiving single and one dual antiplatelet therapy, and four were receiving anticoagulants. All the patients presented an endoscopic pattern of RAVE and were categorized as having a grade B (moderate, 19 patients) or C (severe, five patients) radiation proctopathy [6]. 

Sixteen patients were also receiving medical therapy: seven sucralfate enemas, three topical mesalazine, four topical mesalazine and sucralfate enemas, and two topical corticosteroids. Seven patients were prescribed laxatives (Vaseline oil).

The median number of sessions per patient was two (range = 1–14; mean number = 3.25 ± 3.2). The mean amount of energy released per session was 1591 ± 1344 J. No adverse events, including ulcers, were reported.

Complete remission of bleeding was obtained in 21/24 (88%) patients, while improvement was achieved in another two (global improvement in 23/24, 96%). Of the six patients who were receiving blood transfusions, only one, who underwent a single laser session and then chose to discontinue the treatment, required blood transfusions after treatment (83% resolution of blood transfusion). Remission was stable at the follow-up rectoscopy.

The mean follow-up was 34 ± 26 months.

**Figure 4:**
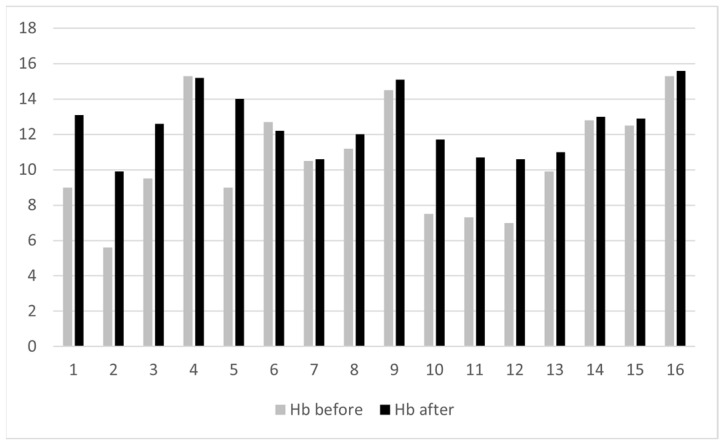
The hemoglobin values of the patients before and after the diode laser treatment.

There was one case (4%) of recurrence after bleeding remission, which was treated successfully with another laser session. Hemoglobin values before and after treatment were available for 16 patients. The hemoglobin levels were significantly higher after treatment (mean and standard deviation 10.6 ± 3.0 before treatment and 12.5 ± 1.7 after treatment, *p* = 0.001) (Figure 4).

The cost of each of the diode laser sessions, which lasted on average a mean of 30 min, was calculated (Table 1). The staff costs are not included in the evaluation, as these vary from hospital to hospital. The materials refer to the personal protective equipment for the surgeon and nurses, a disposable cloth for the patient, a disposable tip for the laser, and the disinfectant and sterilant for cleansing and disinfecting the endoscope. The equipment refers to the PC workstation, colonoscope, laser source device and instruments to cleanse the endoscopic probes.

## 4. Discussion

Currently, radiation therapy is not only the main component of the remedial treatment for several pelvic malignancies, but it is also used as an effective neoadjuvant therapy. A large body of evidence, however, has shown that the application of high doses of radiation to treat malignancies may result in adverse effects on nearby body organs. One of these adverse events is proctitis [1]. Actinic proctopathy affects about 75% of patients undergoing pelvic radiotherapy and becomes chronic (CRP) in 5–20% of cases, with the onset of symptoms some months or even years after the end of the radiation treatment.

Although the rectal mucosa is more resistant to radiation-induced damage than that of the colon and small intestine, the rectum is the segment of the gastrointestinal tract most frequently affected by the actinic insult. This occurs for anatomical reasons, represented both by the fixation of the bowel in the pelvis and by its close proximity to organs whose malignancy is sensitive to radiotherapy, such as the prostate.

CRP is more common in patients who have developed one severe acute actinic proctopathy and in those affected by diabetes, inflammatory bowel disease, hypertension arterial and peripheral vascular diseases. The method of radiation delivery is also an important predictor of the risk for radiation proctitis. 

CRP is characterized by bleeding, diarrhea, fecal urgency and tenesmus. Rarer is the development of stenosis and/or fistulas with adjacent organs. The persistence of symptoms for more than three months, whether preceded or not from an acute onset, defines actinic proctopathy as chronic. Bleeding is the commonest symptom and can occur months or years after radiotherapy following the rupture of superficial vascular lesions developed as a result of tissue ischemia with submucosal fibrosis and obliterative vasculitis [14,15]. CRP results from progressive epithelial atrophy and fibrosis associated with obliterative endarteritis, chronic mucosal ischemia, submucosal fibrosis and new vessel formation, which have been shown to lead to clinical symptoms of bleeding.

The current management of radiation-induced proctitis is frequently carried out in a stepwise mode, moving from less to more invasive strategies [16]. Although there is no endoscopic evidence demonstrating their efficacy, steroids and mesalazine derivatives have led to reports of subjective symptom improvement in patients with mild proctitis. A significant reduction in bleeding, ulcerations and diarrhea was reported in CRP patients after treatment with oral metronidazole together with betamethasone and mesalazine enemas [17]. Sucralfate is thought to play a role in promoting the repair of the epithelium of the colonic mucosa and is also able to form a protective barrier against its damage. Sucralfate enemas are also used for CRP treatment and are particularly safe and well tolerated [18]. Kochhar et al. conducted a prospective, randomized, controlled study in a double-blind study on 37 patients, evaluating the efficacy anti-inflammatory drugs and topical sucralfate. The results of the study, albeit with a follow-up of 4 weeks, showed how the use of a rectal suspension containing two grams of sucralfate, given twice daily for 4 weeks, was able to improve the symptoms of the treated patients with statistical significance [19].

Short-chain fatty acids such as butyric acid represent an important substrate for the oxidative processes of the colonic mucosa, and their mechanism of action could be altered throughout the course of chronic actinic proctopathy. Produced by the fermentation of non-absorbable carbohydrates by intestinal bacteria, short-chain fatty acids also exert a trophic effect on the intestinal mucosa by stimulating proliferation and cellular differentiation. Finally, they are able to dilate the small arterioles of the colonic mucosa as well improve blood flow. Despite these premises, the studies conducted to evaluate the efficacy of topical butyric acid have demonstrated that such a molecule is unable to significantly change the trend of the CRP and its symptoms, except in the short term [20,21]. In cases of severe bleeding, the effect of medical therapy is unfortunately limited [22,23].

Although not innocuous and characterized by local morbidity, according to some studies, local formalin application is an efficacious treatment for hemorrhagic radiation proctitis in 48% of patients [24,25]. Formalin is a mixture of methanol and formaldehyde capable of determining a chemical cauterization of the tissues with which it comes into contact. In the context of CRP, it would seem to act by causing a sclerosis of the fragile newly formed vessels of the mucosa, thereby preventing bleeding. Formalin, usually diluted to 4%, is placed in contact with the rectal mucosa in two ways: by instillation or by gauze soaked in the solution. Formalin instillation was evaluated using different volumes and different times of application (3–10 min) with the patient under local, spinal or general anesthesia, but also not sedated. The number of treatments varies on average from 1.1 to 3.4.

Considering that CRP is the consequence of ischemic damage, hyperbaric oxygen therapy has also been proposed. Hyperbaric oxygen (HBO) therapy enhances the innate healing abilities of a person through the inhalation of 100% oxygen, delivered in daily fractions over a period of weeks via a full body chamber with increased atmospheric pressure. HBO induces the regrowth of damaged vascular endothelial cells in marginally perfused tissue; improves the activity of antioxidant enzymes, thereby reducing free radical damage; and inhibits bacterial overgrowth and toxin production. The side effects of HBO are mild, such as anxiety and acoustic barotraumas, but repeated treatment sessions are necessary; furthermore, efficacy is questionable and the therapy is expensive. Although it can be considered a treatment modality over more invasive procedures, it is not widely available or accepted.

Surgical treatment for CRP is not satisfactory. In fact, fecal diversion has no effect on bleeding and resection is considered an over-treatment, especially bearing in mind the numerous comorbidities of patients [26].

Numerous endoscopic treatments for CRP are reported in the literature. They include potassium titanyl phosphate (KTP) laser, argon laser, neodymium:yttrium–aluminum–garnet (Nd:YAG) laser, heater probe, cryotherapy, radiofrequency ablation (RFA) and argon plasma coagulation (APC) [27,28].

In their respective studies [29,30], Carbatzas and Ventrucci observed how, like other thermal coagulation treatments, the Nd:YAG laser is effective in controlling ongoing rectal bleeding chronic actinic proctopathy, as well as reducing the transfusion requirement of treated patients. The authors report an average of three treatments per patient without evidence of complications. Given the high costs and the development of new methods that are easier to use, the Nd:YAG laser is, however, less used in modern times for the treatment of rectal bleeding during CRP.

The properties of the potassium titanyl phosphate laser are the same as those of the Nd:YAG; however, unlike the latter, the depth of penetration is lower (1–2 mm). Taylor et al. employed the KTP laser in 23 patients with rectal bleeding from CRP; with an average of two treatments for patients (range 1–5), a significant reduction in bleeding as well as an increase in hematocrit levels were obtained. Despite the low depth of tissue penetration by the laser, the authors reported the development of rectal ulcers in two patients, one month after treatment [31].

APC is one of the most utilized by endoscopists [32], who report bleeding remission in 70–90% of patients [33]. The treatment has, however, been associated to a high incidence of adverse events, particularly necrosis and ulcers causing severe bleeding, which has been reported in 47% of cases [34]. APC is a noncontact form of electrocautery that uses ionized argon gas (plasma) to conduct an electrical current from the probe to the nearest grounded tissue. APC superficially burns the rectal mucosa, creating a necrotic layer. During treatment the tissue becomes desiccated, thereby increasing the resistance to the electrical current, thus limiting tissue penetration to approximately 2 to 3 mm. While this ensures the safety of the method during application, it can promote the formation of ulcers in the necrotic tissue [35], and thus, the risk of bleeding recurrence. 

RFA treatment, first introduced for the treatment of Barrett’s esophagus, was subsequently used to treat vascular mucosal lesions, such as gastric antral vascular ectasia (GAVE) or watermelon stomach and CRP. The ablative effect of RFA treatment is restricted to the superficial mucosa, with a lower risk of deep tissue injury. Thanks to the method of application, a wider area of tissue can be treated during a single session, with respect to APC [36,37,38]. RFA probes are, however, more expensive than APCs. 

According to the result of the present study, the diode laser is particularly effective for RAVE. Lasers with long wavelengths of light within the visible spectrum penetrate deeply into the mucosa; thus, the 940 nm diode laser is expected to allow good penetration of the mucosa as a result of its long wavelength. In addition, this wavelength corresponds with the second absorption peak of hemoglobin [39]. Therefore, the 940 nm diode laser has high affinity for hemoglobin. It is then particularly indicated for the treatment of bleeding lesions, such as vascular ectasia. Compared to the Nd:YAG laser, the instrumentation is more compact and comfortable to use. The diode laser also has shallower tissue penetration.

In previous studies, we reported good results in the treatment of chronic radiation proctitis and gastric antral vascular ectasia by the diode laser [8,9]. An affinity between these two entities was hypothesized by Mahmood S et al. [5]. Both diseases are admittedly characterized by the presence of acquired mucosal telangiectasias. 

According to the results of this study, the diode laser achieved complete remission of bleeding in 88% of CRP patients and symptom improvement in 96%. This treatment was also effective in patients requiring blood transfusions and those undergoing antiplatelet and anticoagulant therapy. Laser treatment was also applied to patients with normal hemoglobin values and overt rectal bleeding, in order to improve their quality of life and reduce the risk of anemia. It resulted in a safe procedure with a low risk of complications. In fact, the 940 nm diode laser is characterized by good coagulation properties, easy application and does not penetrate deeply into other layers of tissue [12]. Since we only used non-contact fibers at the 30 W power setting to whiten the vascular lesions, the risk of tissue necrosis and mucosal erosions was avoided in the patients studied here. This feature makes the treatment particularly safe and carries less risk of ulcerations. In fact, we found that it was unnecessary to suspend our patients’ anticoagulant or antiplatelet therapy. Patients with CRP are generally elderly and often have numerous comorbidities, especially cardiovascular disease. Therefore, they are often in chronic treatment with antiplatelet drugs and anticoagulants. It is often the use of these drugs that promotes bleeding in patients with CRP. The use of a treatment that does not necessarily require the suspension of anticoagulant and antiplatelet drugs is advantageous for the management of these patients. Another advantage of diode laser treatment is its safe applicability in an outpatient setting, without the need for hospitalization or sedation, which is particularly important in frail patients. The result of this study confirms on a wider scale what was previously reported [8]. In addition, the present study demonstrates that diode-laser treatment for CRP is cost-effective.

The possibility of treating these patients in an outpatient setting, without sedation or hospitalization, also makes it possible to reduce treatment costs. According to the data collected for the current study, the average cost for the diode laser procedure—which can be performed in an outpatient setting, does not require sedation and lasts in mean only 30 min—is relatively low.

## 5. Conclusions

In conclusion, the data collected and analyzed here confirm that diode laser treatment is a good option for the treatment of bleeding CRP. It can achieve remission of bleeding in most patients, with a low risk of complications. According to the present study, diode laser treatment of bleeding CRP is cost-effective. In fact, since it is possible to treat patients without the need for sedation and hospitalization, treatment costs are reduced. Further prospective studies on large case series are warranted to confirm these results.

## Figures and Tables

**Table 1:** The cost of diode laser treatment for a single session and for a treatment cycle (mean of 3.32 sessions). Additional costs include use of outpatient room and administration costs.

	Total Mean Cost for Session (EUR)	Total Mean Cost for Treatment Cycle (EUR)
Materials	329.86	
Equipment	4.43	
Additional costs	139.11	
Total	473.4	1572

## Data Availability

All data are available in an available archive.

## Supplementary Materials

The following supporting information can be downloaded at: https://www.mdpi.com/article/10.3390/life13041025/s1, Video S1: Laser RAVE—first and third session.

## References

[1] Leiper K., Morris A.I. (2007). Treatment of radiation proctitis. Clin. Oncol..

[2] Placer C., Lizarazu A., Borda N., Elósegui J.L., Navascués J.M.E. (2013). Radiation Proctitis and Chronic and Refractory Bleeding. Experience with 4% Formaldehyde. Cirugía Española.

[3] Haas E.M., Bailey H.R., Farragher I. (2007). Applcation of 10 percent formalin for the treatment of radiation-induced hemorrhagic proctitis. Dis. Colon Rectum.

[4] Takeuchi H., Kimura T., Okamoto K., Aoyagi E., Miyamoto H., Kaji M., Takenaka H., Okamura S., Sato Y., Kato J. (2011). A mechanism for abnormal angiogenesis in human radiation proctitis: Analysis of expression profile for angiogenic factors. J. Gastroenterol..

[5] Mahmood S., Bollipo S., Steele S., Bristow R.G., Choudhury A., Oakland K., Martin J. (2021). It’s All the RAVE: Time to Give up on the “Chronic Radiation Proctitis” Misnomer. Gastroenterology.

[6] Zinicola R., Rutter M.D., Falasco G., Brooker J.C., Cennamo V., Contini S., Saunders B.P. (2003). Haemorrhagic radiation proctitis: Endoscopic severity may be useful to guide therapy. Int. J. Color. Dis..

[7] Ramakrishnaiah V.P.N., Krishnamachari S. (2016). Chronic haemorrhagic radiation proctitis: A review. World J. Gastrointest. Surg..

[8] Polese L., Marini L., Rizzato R., Picardi E., Merigliano S. (2017). Endoscopic diode laser therapy for chronic radiation proctitis. Lasers Med. Sci..

[9] Polese L., Angriman I., Pagano D., Tenderini M.L., Polese F., Frego M., D’Amico D.F., Norberto L. (2006). Laser therapy and surgical treatment in transfusion-dependent patients with upper-gastrointestinal vascular ectasia. Lasers Med. Sci..

[10] World Medical Association Declaration of Helsinki (1997). Recommendations guiding physicians in biomedical research involving human subjects. JAMA.

[11] Laterza L., Cecinato P., Guido A., Mussetto A., Fuccio L. (2013). Management of Radiation-Induced Rectal Bleeding. Curr. Gastroenterol. Rep..

[12] Janda P., Sroka R., Baumgartner R., Grevers G., Leunig A. (2001). Laser treatment of hyperplastic inferior nasal turbinates: A review. Lasers Surg. Med..

[13] Janda P., Sroka R., Tauber S., Baumgartner R., Grevers G., Leunig A. (2000). Diode laser treatment of hyperplastic inferior nasal tubinates. Lasers Surg. Med..

[14] Haboubi N.Y., Schofield P.F., Rowland P.L. (1988). The light and electron microscopic features of early and late phase radiation-induced proctitis. Am. J. Gastroenterol..

[15] Hasleton P.S., Carr N., Schofield P.F. (1985). Vacular changes in radiation bowel disease. Histopatohology.

[16] Garg A.K., Mai W.-Y., McGary J.E., Grant W.H., Butler E.B., Teh B.S. (2006). Radiation proctopathy in the treatment of prostate cancer. Int. J. Radiat Oncol. Biol. Phys..

[17] Cavcić J., Turcić J., Martinac P., Jelincić Z., Zupancić B., Panijan-Pezerović R., Unusić J. (2000). Metronidazole in the treatment of chronic radiation proctitis: Clinical trial. Croat Med. J..

[18] Vanneste B.G.L., Van De Voorde L., De Ridder R.J., Van Limbergen E.J., Lambin P., Van Lin E.N. (2015). Chronic radiation proctitis: Tricks to prevent and treat. Int. J. Color. Dis..

[19] Kochhar R., Patel F., Dhar A., Sharma S.C., Ayyagari S., Aggarwal R., Goenka M.K. (1991). Radiation-induced proctosigmoiditis. Prospective, randomized, double-blind controlled trial of oral sulphasalazine plus rectal steroids versus rectal sucralfate. Dig. Dis. Sci..

[20] Talley N.A., Chen F., King D., Jones M., Talley N.J. (1997). Short-chain fatty acids in the treatment of radiation proctitis: A randomized double-blind, placebo-controlled, cross-over pilot trial. Dis. Colon Rectum.

[21] Pinto A., Fidalgo P., Cravo M., Midões J., Chaves P., Rosa J.C., Birto M.D.A., Leitão C.N. (1999). Short chain fatty acids are effective in short-term treatment of chronic radiation proctitis. Dis. Colon Rectum.

[22] Baum C.A., Biddle W.L., Miner P.B. (1989). Failure of 5-Aminosalicylic acid enemas to improve chronic radiation proctitis. Dig. Dis. Sci..

[23] Kochhar R., Sriram P.V.J., Sharma S.C., Goel R.C., Patel F. (1999). Natural history of late radiation proctosigmoiditis treated with topical sucralfate suspension. Dig. Dis. Sci..

[24] Raman R.R. (2007). Two percent formalin retention enemas for haemorrhagic radiation proctitis: A preliminary report. Dis. Colon Rectum.

[25] Alfadhli A.A., Alazmi W.M., Ponich T., Howard J.M., Prokopiw I., Alaqeel A., Gregor J.C. (2008). Efficacy of Argon Plasma Coagulation Compared with Topical Formalin Application for Chronic Radiation Proctopathy. Can. J. Gastroenterol..

[26] Stuart M., Failes D.G., Killingback M.J., De Luca C. (1980). Irradiation injuries of the large intestine. Dis. Colon Rectum.

[27] Rustagi T., Mashimo H. (2011). Endoscopic management of chronic radiation proctitis. World J. Gastroenterol.

[28] Hanson B., MacDonald R., Shaukat A. (2012). Endoscopic and medical therapy for chronic radiation proctopathy: A systematic review. Dis. Colon Rectum.

[29] Carbatzas C., Spencer G.M., Thorpe S.M., Sargeant L.R., Bown S.G. (1996). Nd:YAG Laser Treatment for Bleeding from Radiation Proctitis. Endoscopy.

[30] Ventrucci M., Di Simone M.P., Giulietti P., De Luca G. (2001). Efficacy and safety of Nd:YAG laser for the treatment of bleeding from radiation proctocolitis. Dig. Liver Dis..

[31] Taylor J.G., DiSario J.A., Bjorkman D.J. (2000). KTP laser therapy for bleeding from chronic radiation proctopathy. Gastrointest. Endosc..

[32] Lenz L., Rohr R., Nakao F., Libera E., Ferrari A. (2016). Chronic radiation proctopathy: A practical review of endoscopic treatment. World J. Gastrointest. Surg..

[33] Swan M.P., Moore G.T.C., Sievert W., Devonshire D.A. (2010). Efficacy and safety of single-session argon plasma coagulation in the management of chronic radiation proctitis. Gastrointest. Endosc..

[34] Pineles D., Hajdu C., Poppers D. (2019). Deep Rectal Ulcer as a Result of Argon Plasma Coagulation Therapy for Radiation Proctopathy. Pract. Gastroenterol..

[35] Hortelano E., Gómez-Iturriaga A., Ortiz-De-Zárate R., Zaballa M., Barturen Á., Casquero F., San-Miguel Í., Carvajal C., Cacicedo J., Del-Hoyo O. (2014). Is argon plasma coagulation an effective and safe treatment option for patients with chronic radiation proctitis after high doses of radiotherapy?. Rev. Esp. Enferm. Dig..

[36] Patel A., Pathak R., Deshpande V., Patel S., Wickremesinghe P., Vadada D. (2014). Radiofrequency ablation using BarRx for the endoscopic treatment of radiation proctopathy: A series of three cases. Clin. Exp. Gastroenterol..

[37] Rustagi T., Corbett F.S., Mashimo H. (2015). Treatment of chronic radiation proctopathy with radiofrequency ablation (with video). Gastrointest. Endosc..

[38] Pigò F., Bertani H., Manno M., Mirante V.G., Caruso A., Conigliaro R.L. (2014). Radiofrequency ablation for chronic radiation proctitis: Our initial experience with four cases. Tech. Coloproctology.

[39] Passeron T., Olivier V., Duteil L., Desruelles F., Fontas E., Ortonne J.-P. (2003). The new 940-nanometer diode laser: An effective treatment for leg venulectasia. J. Am. Acad. Dermatol..

